# On the Employment of Taxis in Strangulated Hernia

**Published:** 1872-02

**Authors:** C. C. F. Gay

**Affiliations:** Buffalo, N. Y., Surgeon of Buffalo General Hospital


### ON THE EMPLOYMENT OF TAXIS IN STRANGULATED HERNIA.

BY C. C. F. GAY, M. D., BUFFALO, N. Y.,
(Surgeon of Buffalo General Hospital.)

Having observed, during the past eighteen months, the progress of several cases of strangulated hernia, in which taxis was successfully employed, I have thought these observations were of sufficient value to the profession to make a record of them.

Whenever a hernia, that is strangulated, is spoken or thought of there is almost always, I think, associated therewith, as necessary to its cure, the use of the knife. But it should be borne in mind, that there are *degrees* of strangulation. One case of strangulation may be so slight in degree as to produce no serious mischief although it may have existed for several days without reduction, while another case may exist that would destroy the life of the patient in from eight to twelve hours, unless relief were obtained at once, since the inflammation would be so active and acute, especially if the patient be plethoric, that the death of the patient would be inevitable unless the knife be used promptly and early. Therefore, in treating strangulated hernia, whether mild or severe in degree, the first thought that will suggest itself to the surgeon in the application of the taxis, will have reference to the *time* that he will be warranted in consuming, in his efforts at reduction.

The time consumed in the use of taxis will, of course, depend upon several circumstances. If the hernia be recent, the surgeon will expect to find a more dangerous condition than he would find had the hernia been old, since in recent hernia, it must be presumed that it had been neglected, perhaps, through ignorance of the patient, until the part had become acutely inflamed, so much so indeed as to preclude the possibility of prolonged taxis, since the slightest manipulations would, perhaps, be inconsistent with the safety or healthfulness of the protruding and inflamed bowel.

Another circumstance existing to determine the time that the surgeon may occupy in his efforts at the reduction of hernia, would be the general condition of his patient. He would examine the frequency and character of the pulse, ascertain the amount of febrile reaction and the state of the patient's stomach. If there were no stercoracious vomiting or other alarming symptoms although the parts protruding were extremely tender to the touch, an effort of from eight to ten minutes would be all the time a surgeon would be waranted in consuming. If not successful at the expiration of this time, he should desist from further present efforts, and make use of the local application of ice, and continue its use, interruptedly, for three or four hours should he find it necessary, or what would, perhaps, be better, before applying ice, deplete the inflamed part by the use of a half-dozen leeches, and afterwards make use of the ice. At the expiration of a few hours, or an hour perhaps, he might be able to return to his patient and reduce the hernia in a moment.

But on the other hand, should the symptoms be more urgent, the pulse rapid and the ejecta from the stomach be of a stercoracious character, prompt action would be required, and the knife used at once.

In case of an old hernia, since the inflammation would be less acute, the patient could be left several days with entire safety, if in the supine position and with ice or some refrigerating lotion, or perhaps, better still, fomentations with cloths rung out of hot water applied over the inflamed parts, but, of course, the duration of the time, when safety would cease and danger begin, must depend upon the general symptoms, together with the amount of local inflammation present. I shall be justified in stating that the judicious surgeon will never operate simply because the hernia is strangulated. The majority of cases may be relieved without incurring the danger of an operation. Although aware, as I am, that this statement conflicts with the views of authors and the teachings of the schools, I think I shall be able to refer to cases that have occurred in my own practice, and observed in the practice of medical friends with whom I have been associated, illustrative of, and pointing clearly to the truthfulness of what I herein assert. No infallible rule can be laid down, having reference to the manner of applying the taxis, or governing the position of the patient. The rules, heretofore suggested, are entirely too arbitrary. The management, of any single case, must stand upon its own individual merits. He who possesses most tact will best succeed. He who has no tact—and there may be such—had better never try to reduce a hernia.

Nothing better than the rules, laid down by authors, if I except rules that are arbitrary, can be suggested, that I am aware of, in reference to the method of employing taxis. I might, however, be allowed simply to suggest, that often it would be wiser, in grasping a hernial tumor for the purpose of its reduction, to *pull* upon it rather than *push* upon it. In other words, while making gentle pressure over and around the tumor with the hand —if the tumor be of sufficient size to be grasped by the hand— gently drawing away the contents from the point of stricture so as to diminish the relative size of the protruding bowel near its exit, at the abdominal ring.

The late lamented Dr. Gibbs, of this State, published an article January 1869, in which he says : "I have been in practice twenty years and have never been compelled to use the knife for the relief of strangulated hernia."

The plan practiced by Dr. Gibbs, was the same as that recommended by Dr. Lentin, a Brazilian surgeon, viz : Rupture of the stricture by introducing the index finger through the stricture and using considerable force in stretching and lacerating it. If this method could be made available and so supercede the necessity of any further study of the anatomical relations of the parts involved and of any further use of the knife, it would certainly be an advance in conservative surgery that would redown to the credit of its author. I think it worthy of trial, as auxiliary to the taxis. Yet, I am quite sceptical as to the utility or safety of the procedure.

Might there not be greater probability of lacerating the bowel than of rupturing the stricture ?

The remark, however, made by Dr. Gibbs, as to the length of time he had practiced his profession without the use of the knife in strangulated hernia, is significant. This remark coming from so wise and skillful a surgeon, as was Dr. Gibbs, should exert—as I doubt not it will—influence for good, by way of deterring from a too precipitate haste in the employment of so dangerous an expedient as the use of the knife.

On January 11th, 1871, I was requested by Dr. Bartlett, of this city, to visit a patient with him, and to go prepared to operate for strangulated femoral hernia.

I found a patient, aged 84 years, with frequent pulse, who had been vomiting stercoracious matter. The hernia was supposed to have been strangulated four days. The hernia was recent, small and upon the left side. There had been much local inflammation, but, with appropriate topical applications, this had been somewhat allayed. Dr. B. and myself, both agreed that, either with or without an operation, the old lady could not live but a few days, and therefore, advised against an operation, leaving responsibility of electing to the friends of the patient, saying to them, that should they request me to operate, I would do so, but only upon condition of such request.

Dr. B. has kindly furnished me with the subsequent history and happy termination of the case. He states that the bowels were open on the twenty-first day after the occurrence of strangulation, and that his patient made a good recovery.

The protrusion, probably for the most part, was omental.

The *position* of the patient is worthy of consideration in all cases where the taxis is employed. In this regard, authors who have heretofore taught that the supine position either upon a hard mattrass or the floor, with the hips elevated, or that the patient should, at times, be turned topsy-turvy, these being positions, prerequisite to success, have failed, I think, to teach the whole truth, as experience and observation demonstrates. There are other positions of the body equally essential to success as those above enumerated. The *upright* position is one of them, and the *semi-prone* position is another.

I have succeed in reducing hernia, after I had failed with the patient placed in all other positions, save the upright and semi-prone positions, by placing the patient upon his side with the thighs flexed upon the body. I have in this position reduced a hernia almost instantaneously, after long trial in other positions. I have never yet succeeded, by turning the patient topsy-turvy, in reducing hernia. I once resorted to this method for experiment, after an operation. The stricture I had divided with the knife; there seemed no obsticle in the way of the return of the bowel, but the bowel did not return, even when the patient was placed almost in the vertical position with the head down.

I have twice or thrice, after the patient's system had been relaxed, taken him by the legs, after the manner taught in the books, dragging him with his head down while his legs were over my shoulders, and have almost exhausted my strength, in this way, to no purpose. Never, in a single instance, have I met with any success by this manner. It is not at all strange that those who have commenced practice with the belief that such a position is proper, should be slow to place any faith in the efficacy of the opposite, or *upright* position. I have a most interesting and instructive case in point, recorded in my note-book, that I will briefly relate. I was called to this case, in consultation, by Professor Wetmore, of this city :— Mrs. P——, aged 42 years, had femoral hernia of right side, of one year's duration, wears a truss, while the truss was left off, the bowel protruded and could not be returned by persistent efforts of Dr. W., while the patient was under the influence of chloroform. This was late in the evening ; local applications were made use of, and the next morning I visited the patient, along with the doctor.

Chloroform was again administered, when both of us failed to reduce the hernia, after a somewhat prolonged trial, with the patient in the supine position. I advised an operation, and during the absence of Dr. W., for chloroform and an assistant, I entered into conversation with the patient upon her own mode of reducing hernia, when she stated that she always, before this, had had no difficulty in reducing it when she was standing in the upright position. I said to her that she should have made this statement before, and requested her to stand up, when, upon slight pressure over the tumor, I felt a gurgling sensation that was evidence of the return of the tumor, and she stated that she felt it returning.

Convinced that the hernia could now be reduced, but not wishing to accomplish it in the absence of Dr. W., as no detriment could accrue to the patient, I requested her to lie down again.

As soon as the doctor returned, she again got up, placing her hand upon the tumor, it was immediately reduced by herself.

In order to make report of this case a little nearer perfect, I should state that the patient, while in the upright position, had a tendency to syncope, and this circumstance undoubtedly facilitated reduction of the hernia, and, perhaps, was as essential an element toward its accomplishment as the position itself. Yet the fact remains that the patient often had occasion to reduce her own hernia, and never could succeed in any other position than the upright one. Whether she always had a tendency to faintness during her manipulations is not known.

During the past summer, I reduced a femoral hernia, right side, by placing my patient upon her right side, nearly in the semi-prone position, with the thighs flexedupon the body; seizing hold of the tumor, I almost immediately reduced it without the aid of chloroform, when I had failed with the patient in almost all other positions, when she was under the influence of chloroform.

I am to consider, therefore, that the positions heretofore recommended by the books are not always the best positions, and that if faintness occur in such positions, then it will be wise to resort to the upright, and if need be, the semi-prone positions, when taxis applied, will be made most serviceable and efficient.

